# The Atlanta Medical Society

**Published:** 1883-04

**Authors:** 


### Societies.

---

### THE ATLANTA MEDICAL SOCIETY.

*Reported for the Atlanta Medical Register.*

ATLANTA, March, 1883.

The President, Dr. W. S. Armstrong, in the chair.

Dr. James B. Baird exhibited a renal calculus of unusual size which was passed by a lady eighty-four years of age. Its passage through the ureter occupied a period of fourteen hours, and was accompanied by intense pain. The diagnosis, verified several days later by the discharge of the stone from the bladder, was made at the time, and opiates and alkaline diuretics were freely administered. The patient has had no subsequent trouble.

Dr. Baird also reported the case of a little girl, aged about eleven years, who suffered from irregular periodical tonic contractions of the muscles of the leg—mainly the gastrocnemius—sometimes in one and sometimes in the other leg. The attacks, from one to three daily and mainly in the night time, lasted from one to three hours. After a trial of hot baths, anodynes, so-called anti-spasmodics, etc., without avail, she was entirely relieved by the faradic current to the affected muscles, and strychnine in doses gradually increased from $\frac{1}{48}$ to $\frac{1}{8}$ of a grain three times daily. She was well nourished, ruddy and apparently otherwise perfectly well. Three months have passed without recurrence of the trouble.

Dr. W. S. Armstrong said that three months ago he operated on a woman, by dilatation and cutting, for fissure of the rectum, who had suffered pain in the back and from the act of defacation for a year.

While she was under ether he discovered a large ulcer, the length of his finger above the anus, on the recto-vaginal septum. The general health of the patient is good. A bare suspicion of syphilis caused him to put her upon mercury and iodide of potassium. No improvement followed, but the ulcer has enlarged and become indurated, and the surrounding parts are somewhat infiltrated. He is suspicious that it is cancerous.

Dr. K. C. Divine asked if the history of the case excluded syphilis, and suggested topical applications.

Dr. V. H. Taliaferro suggested a suppository containing nitrate of silver. He hoped from the symptoms detailed that it was not cancer.

Dr. Baird suggested complete dilatation of the sphincter muscle, thorough exploration, and the topical application of caustic—the solid stick of nitrate of silver, for instance.

Dr. George H. Noble reported a case of stricture of the rectum from dysenteric ulceration, about three and a half inches from the anus, of eighteen years standing.

With difficulty he introduced a uterine dilator about the size of a No. 11 French urethral bougie, with which he gradually dilated until his finger could be forced through the constriction. The dilator was then re-introduced and the intestine put on the stretch, a hernia knife was passed in and several incisions made into the stricture. The resulting hemorrhage was very slight. By gradually increasing the size of the bougies, occasionally inserted, a cure was effected within six months.

A year and a half has elapsed since the operation, yet no appreciable narrowing has recurred.

